# The predictive role of systemic inflammation response index in the prognosis of traumatic brain injury: A propensity score matching study

**DOI:** 10.3389/fneur.2022.995925

**Published:** 2022-11-02

**Authors:** Baojie Mao, Lei Feng, Dongdong Lin, Yanfei Shen, Jiangchun Ma, Yuning Lu, Rui Zhang, Ming Wang, Shu Wan

**Affiliations:** ^1^The Second School of Clinical Medicine, Zhejiang Chinese Medical University, Hangzhou, China; ^2^Brain Center, Affiliated Zhejiang Hospital, Zhejiang University School of Medicine, Hangzhou, China; ^3^Department of Intensive Care, Affiliated Zhejiang Hospital, Zhejiang University School of Medicine, Hangzhou, China

**Keywords:** systemic inflammation response index (SIRI), traumatic brain injury (TBI), all-cause mortality, propensity score matching (PSM), Medical Information Mart for Intensive Care-IV

## Abstract

**Background:**

We aimed to evaluate the predictive power of systemic inflammation response index (SIRI), a novel biomarker, to predict all-cause mortality in patients with traumatic brain injury (TBI) in the intensive care unit (ICU).

**Methods:**

Clinical data were retrieved from the Medical Information Mart for Intensive Care-IV (MIMIC-IV) database. Kaplan-Meier (KM) methods and cox proportional hazard models were performed to examine the association between SIRI and all-cause mortality. The predictive power of SIRI was evaluated compared to other leukocyte-related indexes including neutrophils, lymphocytes, monocytes and white blood cells (WBC) by the Receiver Operating Characteristic (ROC)curve for 30-day mortality. In addition, propensity score matching (PSM) was conducted to reduce confounding.

**Results:**

A total of 350 TBI patients were enrolled overall in our study. The optimal cutoff point of SIRI was determined at 11.24 × 10^9^/L. After 1:1 PSM, 66 matched pairs (132 patients) were generated. During the 30-day, in-hospital and 365-day follow-up periods, patients with low SIRI level were associated with improved survival (*p* < 0.05) compared with patients with high SIRI level. Cox regression analysis identified that higher SIRI values was an independent risk factor for all-cause mortality and results were stable on multiple subgroup analyses. Furthermore, ROC analysis indicated that the area under the curve of SIRI [0.6658 (95% Confidence Interval, 0.5630–0.7687)] was greater than that of neutrophils, monocytes, lymphocytes and WBC. The above results were also observed in the matched cohort.

**Conclusion:**

It was suggested that TBI patients with high SIRI level would suffer from a high risk of 30-day, in-hospital and 365-day mortality. SIRI is a promising inflammatory biomarker for predicting TBI patients' prognosis with relatively better predictive power than other single indicators related to peripheral differential leukocyte counts.

## Introduction

Traumatic brain injury (TBI) has been known as one of the leading causes of disability and death among young adults worldwide, which more than 50 million people suffered from each year (1, 2). The most common external causes of TBI are traffic accidents and falls (3). TBI was commonly categorized into primary and secondary injuries. The primary injury results from mechanical forces that cause direct disruption of the brain tissue. Within several minutes of the primary impact, molecular pathways in the damaged area and surrounding normal tissue are initiated, which play an important role in secondary brain injury (SBI) including excitotoxicity, neuroinflammation and blood-brain barrier (BBB) disruption (4). Rapid CT can be available for identifying almost all forms of TBI and help to make appropriate medical decisions in the shortest time, but little or no information is available by neuroimaging techniques about SBI. Therefore, new biomarkers may be developed for gaining additional information regarding SBI, especially neuroinflammation.

Accumulating evidence have revealed that neuroinflammation plays a critical role in the pathogenesis of SBI. Besides, neutrophils, monocytes and lymphocytes have been thought to be significant working cells in the neuroinflammatory process of traumatic brain injury (2). Therefore, the inflammatory indexes such as neutrophil-to-lymphocyte ratio (NLR) and systemic inflammatory response index (SIRI) are normally thought to represent a crucial predictor of secondary injury. The SIRI is a novel composite indicator with simple detection, strong practicability and low cost. There are reports that SIRI has been shown to have excellent predictive power particularly in cardiovascular disease, infectious diseases and cancer (5–7). In previous studies, it has also been determined to play a great predictive role in prognosis for intracerebral hemorrhage (ICH) and subarachnoid hemorrhage patients (8, 9). However, the association with all-cause death in TBI patients have not been reported yet. This study aimed to evaluate the prognostic significance of SIRI in patients with TBI admitted to the intensive care unit (ICU).

## Materials and methods

### Database

An open and free critical care database, which contained comprehensive clinical data of patients admitted to a tertiary academic medical center in Boston, MA, USA between 2008 and 2019, termed the Medical Information Mart for Intensive Care-IV (MIMIC-IV). The database includes basic patient information, vital signs, laboratory indicators, treatment details and survival data. The information from MIMIC-IV has been approved by the Institutional Review Boards of Beth Israel Deaconess Medical Center (Boston, MA) and Massachusetts Institute of Technology (MIT; Cambridge, MA). As all personal data in this database had been encrypted, informed consent was waived. One author (Mao, Baojie) obtained access to the database and was responsible for data extraction (certification number 46148427).

### Cohort selection

**Figure 1:**
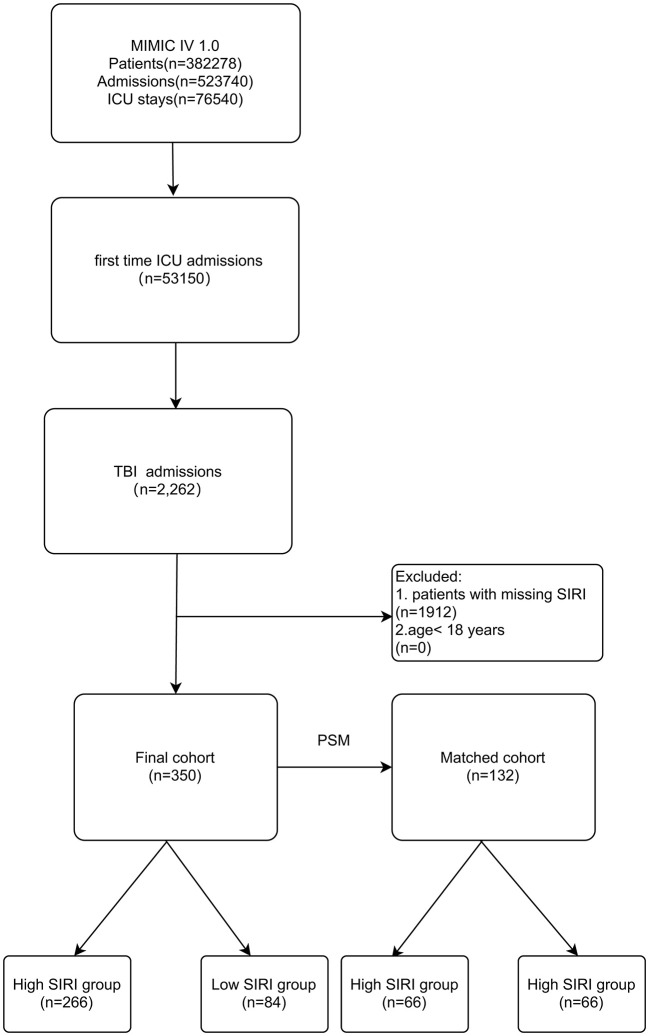
Study flow diagram in the present study.

Those included were patients: (1) first admitted to the ICU during hospital stays; (2) diagnosis of TBI including concussion, cerebral contusion, traumatic epidural hemorrhage, traumatic subdural hemorrhage, traumatic intracerebral hemorrhage and traumatic subarachnoid hemorrhage; (3) age ≥18 years old (Figure 1).

The exclusion criteria were as follows: (1) < 18-year-old; (2) neutrophil counts data, monocyte counts data and lymphocyte counts data at the first day of admission to the ICU were missing; (3) patients with repeated ICU admissions.

### Data exaction

The raw data was obtained by using Structure query language (SQL) with Navicat Premium (version 15) from the MIMIC-IV database. The extracted data included which was collected for the first 24 h in the ICU: (1) baseline variable: age, gender, ethnicity, length of stay in hospital (LOS_hospital), length of stay in ICU (LOS_ICU); (2) vital signs: heart rate, systolic blood pressure (SBP), diastolic blood pressure (DBP), mean blood pressure (MBP), respiratory rate (RR) and temperature; (3) comorbidities: congestive heart failure, diabete, malignant cancer, chronic obstructive pulmonary disease (COPD), hyperlipidemia, liver disease, kidney disease; (4) laboratory events: white blood cell counts, neutrophil counts, monocyte counts, lymphocyte counts, red blood cell counts, platelet counts, hemoglobin, serum sodium, serum potassium, serum creatinine, blood urea nitrogen (BUN), red blood cell distribution width (RDW), prothrombin time (PT), partial thromboplastin time (PTT), international normalized ratio (INR); (5) Systemic Inflammatory Response Syndrome Score (SIRS), Glasgow Coma Scale (GCS), Sequential Organ Failure Assessment score (SOFA), Simplified Acute Physiology Score II(SAPS II) and Acute Physiology Score III (APS III); For some variables measured multiple times within 24 h after ICU admission, their averages were used including heart rate, SBP, DBP, MBP, temperature and RR. For our analysis, all-cause mortality within 30 days was the primary endpoint, while in-hospital and 365-day all-cause death was the secondary endpoint. The SIRI was calculated using the following formula: SIRI = neutrophil counts × monocyte counts/lymphocyte counts.

### Management of missing data

To reduce bias due to missing data, variables with more than 10% missing values were excluded from the study. Correspondingly, few missing values were replaced with overall means.

### Statistical analysis

The Kolmogorov-Smirnov test was used to test for normality distribution. Continuous variables were expressed as mean with standard deviation (for normal distribution) or median with 25–75th percentile (for non-normal distribution), which were analyzed by Student's *t*-test or Mann-Whitney test, as appropriate. Categorical variables were presented as counts (percentages), compared using the chi-square test.

The optimal SIRI cutoff point was obtained through KM curves using R packages “survival” and “survminer.” KM methods and multivariable cox regressions were used to analyze the effects of the SIRI levels and all-cause mortality. Three multivariate analysis models were established for each end point, and the low SIRI level group (< 11.24 × 10^9^ /L) was set as the reference group. In model 1, the covariates were not adjusted; in model 2, the covariates included age, gender and race; in model 3, Los_hospital, platelet, red blood cell, BUN, RR, INR, PT, PTT, temperature, RDW, GCS, SOFA, APSIII, SAPSII, SIRS were further adjusted, with a *P* < 0.1 on univariate analysis. We have also performed a subgroup analysis to determine if the association differed for subgroups classified using different variables including gender, age, comorbidities and various physiological scores. ROC analysis was used to examine the association of admission SIRI with 30-day mortality. The areas under the ROC curves were used to compare the SIRI with other inflammatory indicators. Propensity score matching (PSM) analysis was used to minimize the effect of potential confounders. Confounders in model 3 and baseline imbalanced variables were used to evaluate the propensity scores. PSM was performed at a ratio of 1: 1 using a caliper width of 0.2 of the standard deviation of the logit of the propensity score. *P* < 0.05 were considered statistically significant and all tests were two-tailed. All statistical analyses were performed in R software (version 4.2.0) or STATA software (version 14).

## Results

### Patients characteristics

**Figure 2:**
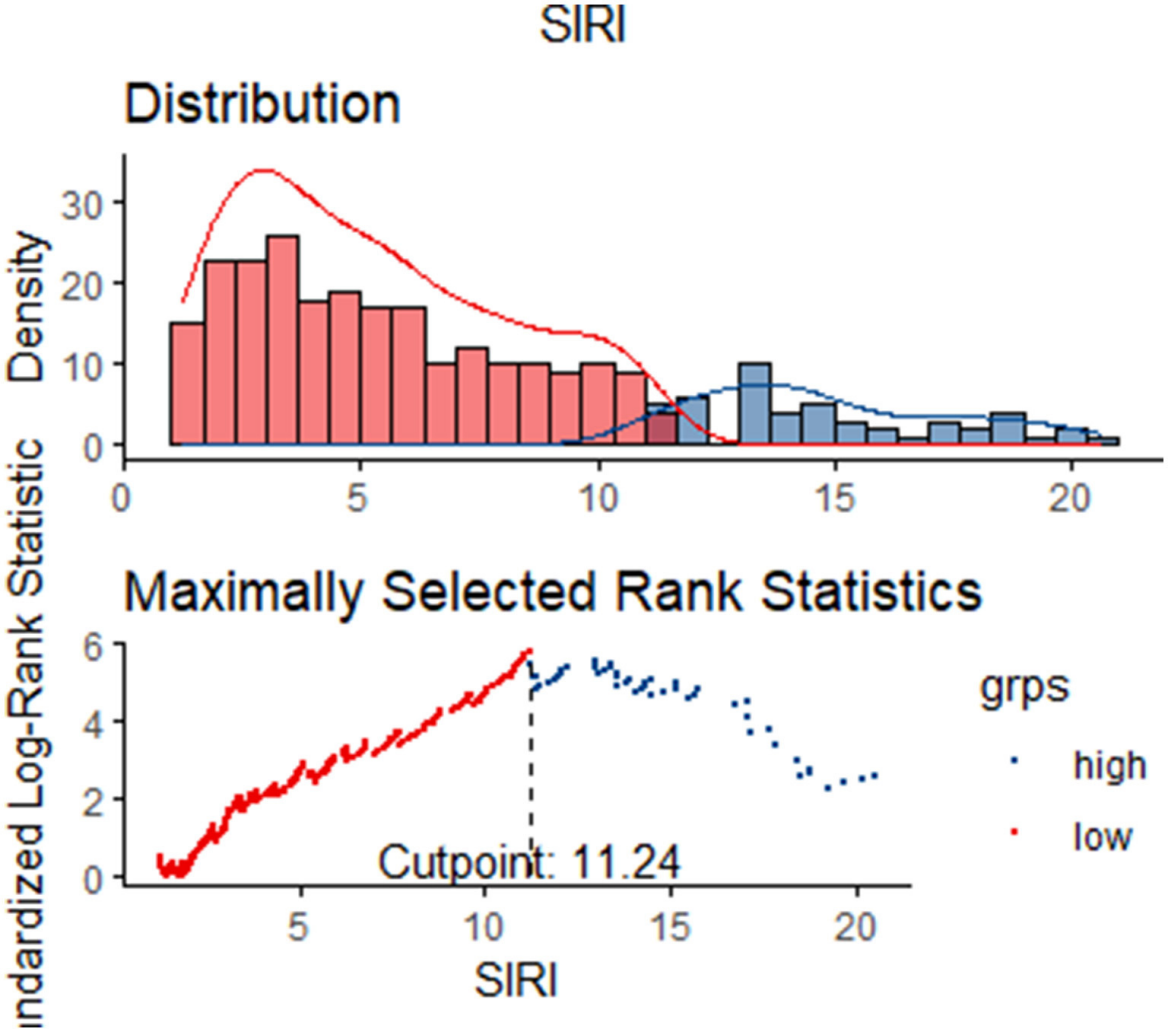
The optimal cutoff value of SIRI.

A total of 350 subjects were analyzed in this study. And after PSM, 66 matched pairs (132 patients) were included as the matched cohort (Table 1). The optimal cutoff value of SIRI determined was 11.24 × 10^9^/L (Figure 2). In the Supplementary Figure 1, The best cutoff value was also confirmed in the restricted cubic splines (RCS). We grouped patients by the measurements of SIRI. In the original cohort, compared to patients with low SIRI (< 11.24 × 10^9^ /L), those with high SIRI (≥ 11.24 × 10^9^ /L) were higher proportion of males (*p* = 0.030), had difference in racial distribution (*p* = 0.043) and higher level of white blood counts (*p* = 0.000), serum creatinine (*p* = 0.012) and BUN (*p* = 0.000). Moreover, the analysis showed that SIRI was associated with an increased risk for all-cause mortality (30-days, 365-days and in-hospital). After PSM, almost all covariates in the matched cohort were well-balanced (*p* > 0.050) between two groups except age, LOS_hospital, LOS_ICU.

**Table 1:** Characteristics of the study patients according to the SIRI groups before and after PSM.

**Characteristic**	**Original cohort**			**Matched cohort**		
	**SIRI < 11.24 × 10^9^**	**SIRI ≥11.24 × 10^9^**	***P***	**SIRI < 11.24 × 10^9^**	**SIRI ≥11.24 × 10^9^**	***P***
N (sample size)	266	84		66	66	
**Baseline variable**	
Age (year)	62.5 (47.0, 77.0)	64.5 (50.0, 79.0)	0.393	55.5 (37.2, 70.0)	65.0 (54.5, 83.0)	0.014
Gender, *n* (%)			0.030			0.533
Male	165 (62.0)	63 (75.0)		53 (80.3)	49 (74.2)	
Female	101 (38.0)	21 (25.0)		13 (19.7)	17 (25.8)	
Ethnicity, *n* (%)			0.043			0.327
White	130 (48.9)	37 (44.0)		21 (31.8)	29 (43.9)	
Black	20 (7.5)	1 (1.2)		2 (3.0)	1 (1.5)	
Other	116 (43.6)	46 (54.8)		43 (65.2)	36 (54.5)	
Los_ICU (day)	4.1 (1.8, 10.4)	3.2 (1.8, 8.8)	0.357	5.5 (2.0, 13.8)	3.0 (1.8, 6.1)	0.010
Los_hospital (day)	12.3 (6.5, 23.9)	10.0 (5.0, 20.1)	0.120	16.3 (7.2, 25.2)	9.6 (4.7, 18.8)	0.015
**Coexisting comorbidities**, ***n*** **(%)**	
Congestive heart failure	39 (14.7)	16 (19.1)	0.336	6 (9.1)	12 (18.2)	0.205
Diabetes	67 (25.2)	22 (26.2)	0.854	13 (19.7)	13 (19.7)	1.000
Malignant cancer	16 (6.0)	3 (3.6)	0.389	2 (3.0)	2 (3.0)	1.000
COPD	18 (6.8)	9 (10.7)	0.237	5 (7.6)	7 (10.6)	0.762
Hyperlipidemia	75 (282)	27 (32.1)	0.488	17 (25.8)	23 (34.8)	0.344
Liver disease	24 (19.0)	6 (7.1)	0.592	4 (6.1)	3 (4.5)	1.000
Kidney disease	59 (22.2)	25 (29.8)	0.156	15 (22.7)	17 (25.8)	0.839
**Laboratory tests**	
WBC,10^9^ g/l	10.7 (7.8, 13.9)	14.2 (11.9, 18.6)	0.000	14.2 (12.1, 16.2)	13.6 (11.7, 16.6)	0.955
Platelet, 10^9^/L	177.0 (131.0, 224.0)	177.5 (136.5, 218.5)	0.964	190.0 (153.5, 233.0)	179.5 (137.5, 218.8)	0.386
Hemoglobin, g/Dl	11.3 (9.6, 12.8)	11.5 (10.1, 13.3)	0.237	12.3 (10.1, 13.6)	11.8 (10.1, 13.3)	0.451
Red blood cell, 10^9^/L	3.7 (3.2, 4.2)	3.8 (3.3, 4.4)	0.248	3.9 (3.3, 4.4)	3.8 (3.3, 4.4)	0.740
Serum creatinine, mg/Dl	0.8 (0.7, 1.1)	0.9 (0.8, 1.3)	0.012	0.9 (0.8, 1.2)	0.8 (0.7, 1.1)	0.580
BUN, mg/dL	14.0 (11.0, 20.0)	18.0 (14.0, 23.0)	0.000	16.0 (12.0, 21.0)	17.0 (14.0, 23.0)	0.430
Serum potassium, mmol/L	4.0 (3.7, 4.5)	4.2 (3.0, 4.6)	0.212	4.1 (3.8, 4.5)	4.2 (3.8, 4.5)	0.825
Serum sodium, mmol/L	139.0 (136.0, 142.0)	140.0 (138.0, 142.0)	0.378	139.0 (136.0, 142.0)	140.0 (138.0, 142.0)	0.077
RDW, %	13.7 (13, 14.8)	13.7 (13, 15.0)	0.819	13.4 (12.9, 14.6)	13.5 (12.7, 14.6)	0.077
INR	1.2 (1.1, 1.3)	1.2 (1.1, 1.3)	0.509	1.2 (1.1, 1.3)	1.2 (1.1, 1.3)	0.991
PT, s	12.7 (11.7, 14)	12.8 (11.8, 14.5)	0.525	12.8 (11.6, 13.9)	12.8 (11.9, 13.9)	0.736
PTT, s	27.3 (25.3, 29.8)	27.8 (25.7, 29.7)	0.685	27.6 (25.4, 29.6)	27.3 (25.6, 29.1)	0.831
**Vital sign**	
Heart rate, beats/minute	83.0 (72.3, 93.6)	80.6 (72.8, 93.4)	0.786	87.3 (76.9, 95.8)	79.8 (74.3, 91.6)	0.129
SBP, mmHg	122.4 (112, 132.8)	124.5 (113.8, 130.5)	0.754	122.0 (111.7, 132.9)	124.3 (116.1, 130.0)	0.514
DBP, mmHg	65.9 (58.9, 74.9)	63.3 (58.6, 71.2)	0.059	66.0 (61.2, 73.1)	64.1 (58.9, 73.0)	0.231
Temperature, °C	37.0 (36.8, 37.4)	37.0 (36.8, 37.4)	0.621	37.2 (36.8, 37.6)	37.1 (36.9, 37.4)	0.610
MBP, mmHg	81.9 (75.1, 89.7)	80.0 (73.2, 87.4)	0.074	81.5 (75.9, 89.2)	81.0 (74.2, 87.6)	0.423
RR, times/minute	18.2 (16.5, 20.4)	18.4 (16.9, 21.2)	0.371	19.0 (17.0, 21.0)	18.5 (17.3, 21.2)	0.566
Scoring systems	
SIRS	3.0 (2.0, 3.0)	3.0 (2.0, 3.0)	0.221	3.0 (2.0, 4.0)	3.0 (2.0, 3.0)	0.450
GCS	13.0 (9.0, 14.0)	13.0 (7.0, 14.0)	0.192	12.0 (7.2, 14.0)	13.0 (8.2, 14.0)	0.878
SOFA	4.0 (3.0, 7.0)	5.0 (3.0, 8.0)	0.205	5.0 (4.0, 8.0)	5.0 (3.0, 7.0)	0.437
APSIII	41.0 (31.0, 55.0)	45.0 (34.0, 59.5)	0.132	44.0 (32.5, 59.0)	44.0 (32.5, 52.0)	0.574
SAPSII	33.0 (27.0, 40.0)	35.5 (26.5, 41.0)	0.399	33.0 (24.8, 38.8)	35.5 (26.2, 40.8)	0.425
**Clinical outcomes**, ***n*** **(%)**	
30-day mortality	17 (6.4)	25 (29.8)	0.000	2 (3.0)	19 (28.8)	0.000
365-day mortality	25 (9.4)	26 (31.0)	0.000	5 (7.6)	20 (30.3)	0.002
In-hospital mortality	21 (7.9)	26 (29.8)	0.000	4 (6.1)	20 (30.3)	0.001

SIRI, systemic inflammation response index; LOS_hospital, length of stay in hospital; LOS_ICU, length of stay in ICU; WBC, white blood cell; RDW, red blood cell distribution width; SIRS, systemic inflammatory response syndrome score; SOFA, Sequential Organ Failure Assessment; GCS, Glasgow Coma Scale; SAPS II, Simplified Acute Physiology Score II; APS III, Acute Physiology Score III; SBP, systolic blood pressure; DBP, diastolic blood pressure; MBP, mean blood pressure; COPD, chronic obstructive pulmonary disease; PT, prothrombin time; PTT, partial thromboplastin time; INR, international normalized ratio; BUN, blood urea nitrogen; RR, respiratory rate.

### Prognostic significance of SIRI for all-cause mortality

**Figure 3:**
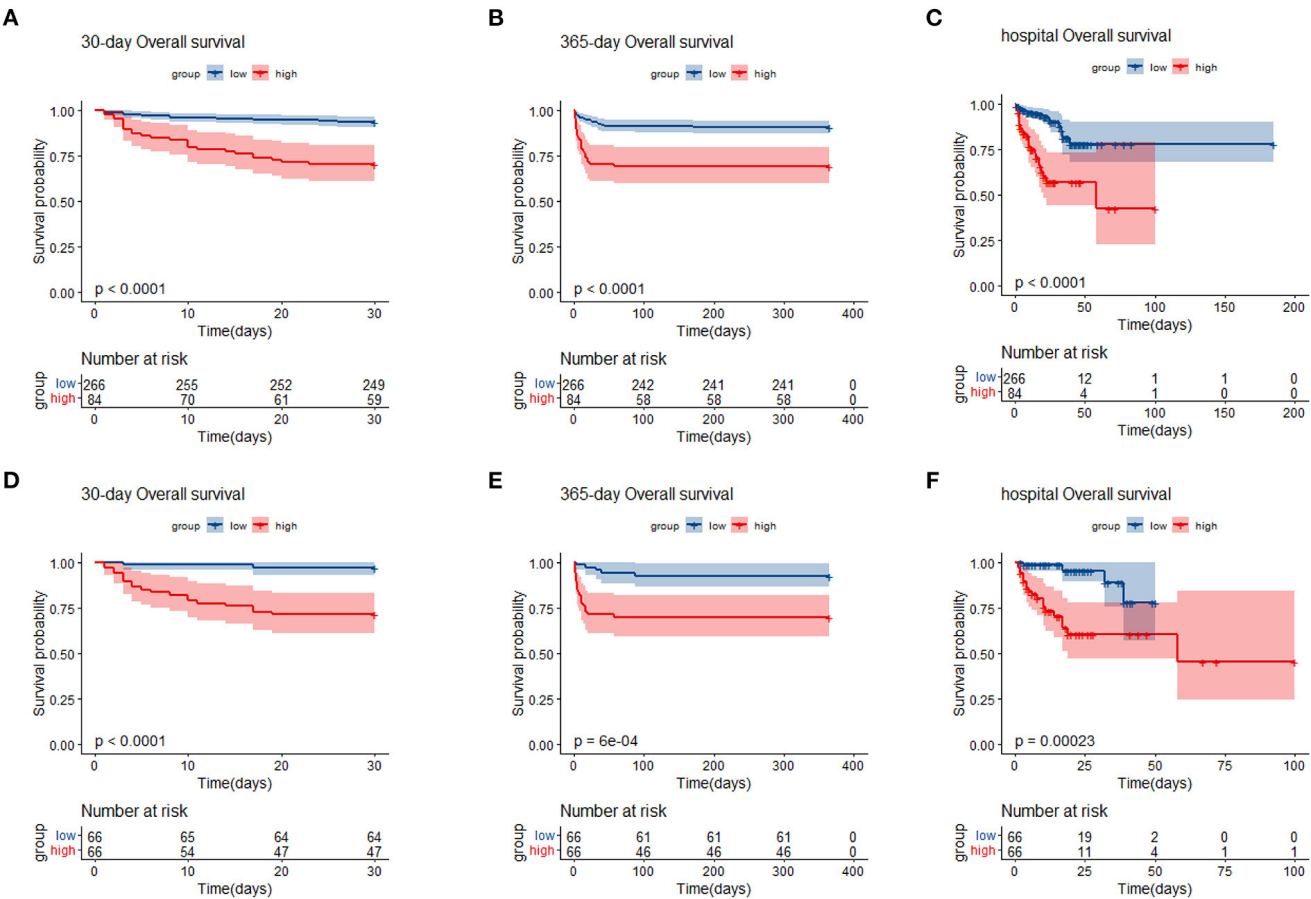
Kaplan–Meier survival analysis plot for 30-day, 365-day survival and in-hospital in patients before PSM **(A–C)**, and patients after PSM **(D–F)**. SIRI, systemic inflammation response index; PSM, propensity score matching; group low (< 11.24 × 10^9^/L); group high (≥11.24 × 10^9^/L).

We generated KM curves for the different groups. Before PSM, 12.0% (42/350) died during the first 30 days, 13.4% (47/350) died during the hospital period, and 14.9% (52/350) died during the 365-day follow-up period. Among the 132 TBI patients included after PSM, 15.9% (21/132) died during the first 30 days, 18.9% (25/132) died during the hospital period, and 18.1% (24/132) died during the 365-day follow-up period (Table 1). As was shown in the Figure 3, patients with low SIRI level were associated with improved survival (*p* < 0.050) compared with patients with high SIRI level during the 30-day, in-hospital and 365-day follow-up periods both before and after PSM.

Different cox proportional hazard regression models were developed to assess the relationship between SIRI and prognosis in TBI patients simultaneously adjusting for possible covariates. The results of these relationships are shown in Table 2. For 30-day all-cause mortality, HR (95% CI) of high SIRI level group (≥11.24 × 10^9^ /L) was 5.31 (2.86, 9.83) in unadjusted model compared with low SIRI level group (< 11.24 × 10^9^ /L). After adjusting for age, gender and race, the association still existed (*P* < 0.05), and the HR (95% CI) was 5.03 (2.67, 9.44). Further adjustment of possible covariates whose *P* < 0.1 on univariate analysis showed similar correlation in model 3 (*P* < 0.05), and the HR (95% CI) was 3.74 (1.87, 7.47). A similar correlation was observed between 365-day and in-hospital all-cause mortality. After PSM, Cox proportional hazard regression analysis revealed that low SIRI level was independently related to better prognosis of TBI patients (Table 3).

**Table 2:** Association between SIRI and clinical outcomes of critically ill patients with TBI before PSM.

**Clinical outcomes**	**Original cohort**					
	**Model 1**		**Model 2**		**Model 3**	
	**HR (95%Cl)**	***P***	**HR (95%Cl)**	***P***	**HR (95%Cl)**	***P***
**Primary outcome**
30-day mortality
Continuous variable	1.02 (1.01, 1.04)	0.001	1.02 (1.01, 1.04)	0.005	1.03 (1.01, 1.05)	0.001
Categorical variables
SIRI < 11.24	1		1		1	
SIRI ≥ 11.24	5.31 (2.86, 9.83)	0.000	5.03 (2.67, 9.44)	0.000	3.74 (1.87, 7.47)	0.000
**Secondary outcomes**
365-day mortality
Continuous variable	1.02 (1.01, 1.04)	0.005	1.02 (1.00, 1.03)	0.020	1.03 (1.01, 1.04)	0.001
Categorical variables
SIRI < 11.24	1		1		1	
SIRI ≥ 11.24	3.85 (2.22, 6.67)	0.000	3.65 (2.08, 6.40)	0.000	3.41 (1.87, 6.22)	0.000
In-hospital mortality
Continuous variable	1.02 (1.01, 1.04)	0.002	1.02 (1.01, 1.04)	0.003	1.03 (1.00, 1.05)	0.018
Categorical variables
SIRI < 11.24	1		1		1	
SIRI ≥ 11.24	4.34 (2.44, 7.73)	0.000	4.15 (2.31, 7.45)	0.000	2.25 (1.04, 4.85)	0.038

Cox proportional hazards regression models were used to calculate hazard ratios (HR) with 95% confidence intervals (CI). Model 1 covariates were adjusted for nothing. Model 2 covariates were adjusted for age, sex and ethnicity. Model 3 covariates were adjusted for Los_hospital, platelet, red blood cell, BUN, RR, INR, PT, PTT, temperature, RDW, GCS, SOFA, APSIII, SAPSII, SIRS.

**Table 3:** Association between SIRI and clinical outcomes of critically ill patients with TBI after PSM.

**Clinical outcomes**	**Matched cohort**					
	**Model 1**		**Model 2**		**Model 3**	
	**HR (95%Cl)**	***P***	**HR (95%Cl)**	***P***	**HR (95%Cl)**	***P***
**Primary outcome**	
30-day mortality	
Continuous variable	1.03 (1.01, 1.05)	0.005	1.02 (1.00, 1.04)	0.014	1.04 (1.02, 1.07)	0.002
Categorical variables	
SIRI < 11.24	1		1		1	
SIRI ≥ 11.24	11.00 (2.56, 47.25)	0.001	9.91 (2.26, 43.45)	0.002	7.78 (1.58, 38.17)	0.012
**Secondary outcomes**	
365-day mortality	
Continuous variable	1.02 (1.01, 1.04)	0.005	1.02 (1.00, 1.04)	0.035	1.03 (1.01, 1.05)	0.013
Categorical variables	
SIRI < 11.24	1		1		1	
SIRI ≥ 11.24	4.74 (1.78, 12.65)	0.002	4.30 (1.57, 11.74)	0.004	4.20 (1.43, 12.35)	0.009
In-hospital mortality	
Continuous variable	1.02 (1.01, 1.04)	0.007	1.02 (1.00, 1.04)	0.034	1.03 (0.99, 1.07)	0.198
Categorical variables	
SIRI < 11.24	1		1		1	
SIRI ≥ 11.24	5.99 (2.03, 17.66)	0.001	5.35 (1.77, 16.21)	0.003	2.46 (0.32, 18.75)	0.385

Cox proportional hazards regression models were used to calculate hazard ratios (HR) with 95% confidence intervals (CI). Model 1 covariates were adjusted for nothing. Model 2 covariates were adjusted for age, sex and ethnicity. Model 3 covariates were adjusted for Los_hospital, platelet, red blood cell, BUN, RR, INR, PT, PTT, temperature, RDW, GCS, SOFA, APSIII, SAPSII, SIRS.

### Subgroup analyses for 30-day mortality

In order to verify the robustness and consistency of our findings, we performed subgroup analyses to assess the association between SIRI and 30-day mortality (Table 4). The result showed the higher SIRI was linked to deteriorative mortality in most strata except in female patients (*p* = 0.055) and patients with hyperlipidemia (*p* = 0.058) or high GCS (*p* = 0.053). Additionally, no statistically significant interactions between SIRI and subgroups were observed. Taken together, the above outcomes prove that results were relatively stable in TBI patients.

**Table 4:** Subgroup analysis of the associations between 30-day all-cause mortality and the SIRI.

	**No. of patients**	**HR (95% CI)**	***P*-value**	***P* for interaction**
Age				0.579
≥63	178	4.49 (2.05, 9.82)	0.000	
< 63	172	5.20 (1.85, 14.60)	0.002	
Gender				0.147
Male	228	7.24 (3.16, 16.60)	0.000	
Female	122	2.93 (0.98, 8.74)	0.055	
Diabetes				0.279
Yes	89	11.48 (2.95, 44.69)	0.000	
No	261	4.12 (2.00, 8.48)	0.000	
Congestive heart failure				0.377
Yes	55	14.02 (2.73, 72.04)	0.002	
No	295	4.12 (2.06, 8.24)	0.000	
Hyperlipidemia				0.558
Yes	102	4.07 (0.95, 17.38)	0.058	
No	248	5.36 (2.63, 10.96)	0.000	
Kidney disease				0.387
Yes	266	5.35 (2.48, 11.55)	0.000	
No	84	3.01 (1.00, 9.05)	0.049	
GCS				0.089
≥13	191	2.53 (0.99, 6.47)	0.053	
< 13	151	9.07 (3.52, 23.34)	0.000	
SOFA				0.562
≥5	180	4.48 (2.21, 9.05)	0.000	
< 5	170	7.72 (1.75, 34.05)	0.007	
SAPSII				0.985
≥34	180	4.80 (2.32, 9.91)	0.000	
< 34	170	4.41 (1.29, 15.05)	0.018	
APSIII				0.394
≥42	182	3.90 (1.99, 7.64)	0.000	
< 42	168	7.12 (1.25, 40.48)	0.027	
RDW				0.636
≥13.7	171	6.29 (2.98, 13.29)	0.000	
< 13.7	179	4.52 (1.35, 15.10)	0.014	

Confounders adjustment were performed as in Model 2. Cox proportional hazards regression models were used to calculate hazard ratio (HR) with 95% confidence interval (CI). The low SIRI level group (< 11.24 × 10^9^ /L) was set as the reference group.

### ROC curve analysis for 30-day mortality

**Figure 4:**
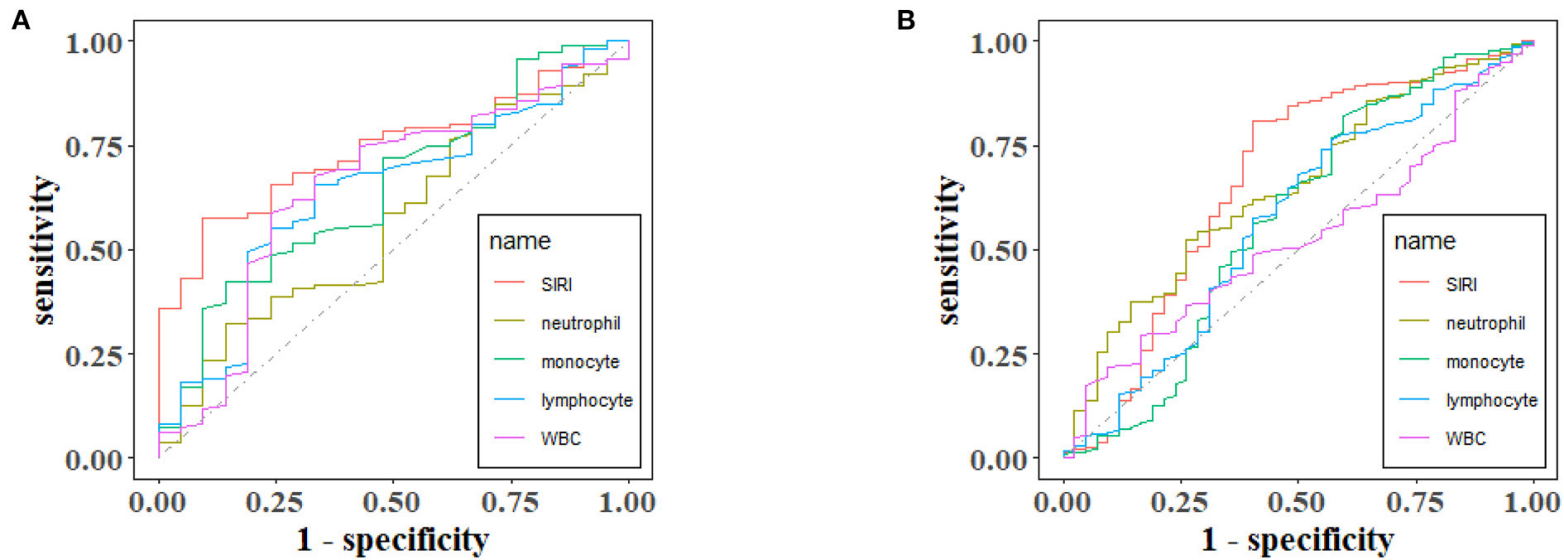
The receiver operating characteristic (ROC) curves of predictive value of inflammatory indexes for 30-day mortality in TBI before **(A)** and after **(B)** PSM.

ROC curves were plotted to assess the usefulness of SIRI, neutrophils, lymphocytes, monocytes and WBC in predicting mortality in TBI patients. We found that SIRI was relatively more accurate than other single inflammatory markers including neutrophils, lymphocytes, monocytes and WBC (AUC 0.6658 vs. 0.6644; 0.6658 vs. 0.4762; 0.6658 vs. 0.6607; 0.6658 vs. 0.5824, respectively) in the original cohort (Figure 4A and Table 5). Besides, analyses using PSM yielded essentially the same results (AUC 0.7435 vs. 0.5654; 0.7435 vs. 0.6489; 0.7435 vs. 0.6379; 0.7435 vs. 0.6499, respectively) in the Figure 4B, Table 6.

**Table 5:** Receiver operating curve (ROC) for prediction for 30-day mortality in TBI Patients before PSM.

	**ROC area (AUC)**	**95% CI low**	**95% CI upp**
SIRI	0.6658	0.5630	0.7687
Neutrophils	0.6489	0.5617	0.7361
Monocytes	0.5752	0.4655	0.6849
Lymphocytes	0.5698	0.4693	0.6703
WBC	0.5259	0.4401	0.6116

SIRI, systemic inflammation response index; WBC, white blood cell.

**Table 6:** Receiver operating curve (ROC) for prediction for 30-day mortality in TBI patients after PSM.

	**ROC area (AUC)**	**95% CI low**	**95% CI upp**
SIRI	0.7435	0.6500	0.8369
Neutrophils	0.5654	0.4331	0.6978
Monocytes	0.6489	0.5218	0.7760
Lymphocytes	0.6379	0.5105	0.7654
WBCs	0.6499	0.5148	0.7850

SIRI, systemic inflammation response index; WBC, white blood cell.

## Discussion

In the current study enrolled 350 TBI patients admitted to the ICU, we found that patients with high SIRI level were associated with a significantly increased risk of all-cause mortality and demonstrated that SIRI was an independent predictor of all-cause in TBI patients after adjusting confounding factors.

As an easily available biomarker, SIRI is an emerging systemic inflammatory marker based on peripheral neutrophils, monocytes, and lymphocytes, which first was developed in 2016 (10). In recent years, scholars have found that SIRI can be used as an indispensable indicator of leukocyte subtypes in neurological diseases such as glioblastoma, acute ischemic stroke and hypoxic ischemic encephalopathy (11–13). Besides, elevated SIRI index could be independent predicting factors for poor outcomes among ICH and aneurysmal subarachnoid hemorrhage patients (8, 9, 14).

To our knowledge, this is the first study to investigate the relationship between SIRI and all-cause mortality in a severe TBI population. First, Analysis of baseline characteristics showed that the mortality of TBI patients was statistically significant be between different SIRI levels (*p* < 0.05).We found that higher SIRI (≥11.24 × 10^9^/L) was associated with a higher risk of all-cause mortality at 30 days, 365 days, and during the hospital period in our recent study. Then, cox regression analyses revealed that a higher admission SIRI value was an independent risk factor for 30-day, 365-day and in-hospital mortality. In the matched cohort, there is no statistical significance between SIRI and in-hospital mortality in model 3, which may be due to the excessive weight of hospital stays (*p* = 0.385). Besides, a series of subgroup analyses were performed to verify the robustness of our findings. As shown in subgroup analyses, SIRI maintained its predictive power in most strata. Furthermore, ROC curves revealed that the AUC of admission SIRI was better than those of neutrophils, lymphocytes, monocytes and WBC. After PSM, The AUC of admission SIRI was >0.7, indicating that SIRI has considerable predictive value for 30-day mortality.

More and more studies have demonstrated the correlation of inflammation in the pathogenesis of TBI (2, 15). Secondary damage due to TBI induced by inflammatory cells and inflammatory cascades plays a key role in progression of disease, thus affecting clinical prognosis. Neutrophils are the first responders to tissue injuries in the central nervous system (CNS), followed by monocytes, lymphocytes, and mast cells. On the one hand, neutrophils play a critical role in controlling damage lesions and clearing cellular debris and damaged cells (16). On the other hand, neutrophils are not always neuroprotective and have the ability to break down the BBB and promote neuronal cells death by releasing various inflammatory factors (2). Similarly to neutrophils, monocytes recruitment in circulating and damaged tissues are known to be a characteristic hallmark of inflammation (17). Monocyte-derived macrophages were found to be crucial for the chronic phase that occurred several weeks after brain injury on animal models (2). Previous studies have shown that a higher monocyte count was independently associated with poor outcomes among intracerebral hemorrhage patients (18–20), which supports our research to some extent. When it comes to lymphocytes, the role in patients with brain injury remains to be studied in the future. ROS release can promote T lymphocytes recruitment by activating endothelial barriers (21). In addition, T cells play no significant role in early TBI pathogenesis (22).

It should be noted that our study had some limitations that may prevent the generalizability of findings. First, subgroup analysis was made according to factors which were reported to have strong relationship with SIRI. However, the stability of these results may be limited due to the small sample size. Second, some important data, such as cranial surgery, severity of intracranial pathology on CT, were missing in this database. Aiming to alleviate the impact of disease severity, we have included GCS, SOFA, and APS III in the PSM analysis, which to a certain degree can also reflect the severity of brain injury. However, bias risk due to disease severity still needs to be considered. Third, consistent with previous studies, only the first SIRI on ICU admission was calculated. It is worth noting that the dynamic changes in SIRI may also have a significant correlation with prognosis in TBI, which still needs to be further investigated. Moreover, although a non-linear association trend was found in the RCS analysis, it remains non-significant, especially in high SIRI values, which may be due to the small sample size. Besides, the number of covariates associated with TBI prognosis is very large and under-collected in our study. Finally, patients with missing data were excluded from this analysis, which could have biased the results.

## Conclusion

This study provides an easy-to-get biomarker for predicting prognosis in patients with TBI admitted to the ICU. Patients with high SIRI level would suffer from a high risk of all-cause mortality. SIRI is a promising composite inflammatory biomarker for predicting TBI prognosis with relatively better predictive power than single inflammatory biomarkers—neutrophils, lymphocytes, monocytes and WBC.

## Data availability statement

The raw data supporting the conclusions of this article will be made available by the authors, without undue reservation.

## Ethics statement

The studies involving human participants were reviewed and approved by Institutional Review Boards of Beth Israel Deaconess Medical Center (Boston, MA) and Massachusetts Institute of Technology (MIT; Cambridge, MA). Written informed consent for participation was not required for this study in accordance with the national legislation and the institutional requirements.

## Author contributions

SW and MW designed the work. BM, YS, DL, and JM extracted and analyzed the datasets. BM and LF wrote this paper. BM, RZ, and YL interpreted the results and helped to revise the manuscript. All authors read and approved the final manuscript.

## Funding

This work was supported by the grants from Medical Health Science and Technology Key Project of Zhejiang Provincial Health Commission (WKJ-ZJ-2014), Key Research and Development Project of Zhejiang Provincial Department of Science and Technology (2021C03105), and Provincial Natural Science Foundation of Zhejiang (Y21H090041).

## Conflict of interest

The authors declare that the research was conducted in the absence of any commercial or financial relationships that could be construed as a potential conflict of interest.

## Publisher's note

All claims expressed in this article are solely those of the authors and do not necessarily represent those of their affiliated organizations, or those of the publisher, the editors and the reviewers. Any product that may be evaluated in this article, or claim that may be made by its manufacturer, is not guaranteed or endorsed by the publisher.

## Abbreviations

TBI: traumatic brain injury
SIRI: systemic inflammation response index
SBI: secondary brain injury
NLR: neutrophil-to-lymphocyte ratio
ICH: intracerebral hemorrhage
RDW: red blood cell distribution width
SIRS: Systemic Inflammatory Response Syndrome Score
SOFA: Sequential Organ Failure Assessment
SAPS II: Simplified Acute Physiology Score II
GCS: Glasgow Coma Scale
APSIII: Antiphospholipid syndrome
ROC: Receiver Operating Characteristic
SBP: systolic blood pressure
DBP: diastolic blood pressure
MBP: mean blood pressure
COPD: chronic obstructive pulmonary disease
PSM: propensity score matching
HR: hazard ratios
CI: confidence intervals
BBB: blood brain barrier
ICU: intensive care unit
PT: prothrombin time
PTT: partial thromboplastin time
INR: international normalized ratio
BUN: blood urea nitrogen
LOS_hospital: length of stay in hospital
LOS_ICU: length of stay in ICU
RR: respiratory rate
CNS: central nervous system
WBC: white blood cell
RCS, restricted cubic splines, KM: Kaplan-Meier.
