# Supplementary material for: The predictive role of systemic inflammation response index in the prognosis of traumatic brain injury: A propensity score matching study
Source: Front Neurol. 2022 Nov 2;13:995925. doi: 10.3389/fneur.2022.995925 (PMC9666699; doi:10.3389/fneur.2022.995925)
Supplement: Supplementary file 1 [file Data_Sheet_1.PDF]

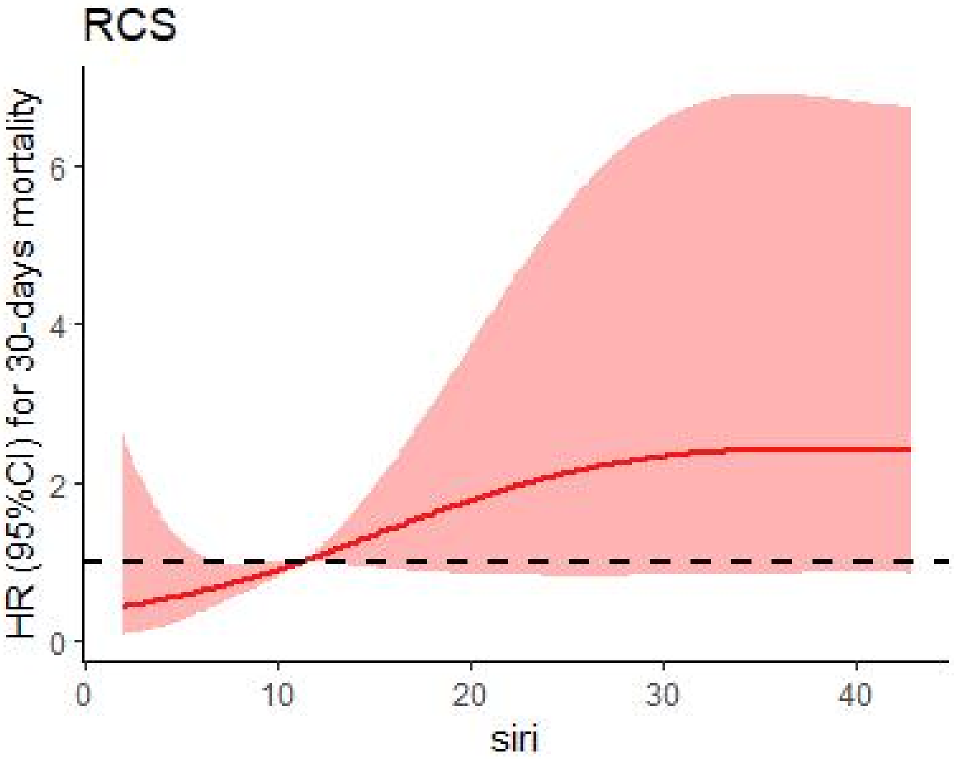

FIGURE 1 Association of admission SIRI with 30-day mortality risk. HR is indicated by the solid red line, and 95% CI is denoted by the shaded red area.

TABLE 1 SIRI-HR TABLE for 30-day mortality from RCS.

SIRI	HR
1.596628	0.002043
1.784722	0.002484
1.972817	0.003018
2.160911	0.003666
2.349005	0.004451
2.5371	0.005399
2.725194	0.006543
2.913288	0.007921
3.101383	0.009575
3.289477	0.011558
3.477571	0.013928
3.665666	0.016751
3.85376	0.020104
4.041854	0.024073
4.229949	0.028754
4.418043	0.034254
4.606138	0.040689
4.794232	0.048185

4.982326	0.056876
5.170421	0.066905
5.358515	0.078417
5.546609	0.091559
5.734704	0.106475
5.922798	0.123302
6.110892	0.142162
6.298987	0.163156
6.487081	0.186358
6.675175	0.211804
6.86327	0.239486
7.051364	0.26934
7.239458	0.301241
7.427553	0.334995
7.615647	0.370333
7.803741	0.406987
7.991836	0.444712
8.17993	0.483244
8.368024	0.522299
8.556119	0.561588
8.744213	0.600812
8.932308	0.639677
9.120402	0.677895
9.308496	0.715191
9.496591	0.751307
9.684685	0.786008
9.872779	0.819085
10.06087	0.850358
10.24897	0.879677
10.43706	0.906926
10.62516	0.932022
10.81325	0.954913
11.00135	0.975581
11.18944	0.994038
11.37753	1.010325
11.56563	1.024509
11.75372	1.036682
11.94182	1.046955
12.12991	1.055461
12.31801	1.062345
12.5061	1.067768
12.69419	1.0719
12.88229	1.07492
13.07038	1.077011

13.25848	1.078364
13.44657	1.079168
13.63467	1.079619
13.82276	1.079909
14.01085	1.080199
14.19895	1.080519
14.38704	1.080868
14.57514	1.081246
14.76323	1.081654
14.95133	1.082091
15.13942	1.082556
15.32752	1.08305
15.51561	1.083572
15.7037	1.084122
15.8918	1.084701
16.07989	1.085307
16.26799	1.085941
16.45608	1.086602
16.64418	1.087291
16.83227	1.088007
17.02036	1.088749
17.20846	1.089519
17.39655	1.090315
17.58465	1.091138
17.77274	1.091987
17.96084	1.092862
18.14893	1.093763
18.33702	1.094689
18.52512	1.095642
18.71321	1.09662
18.90131	1.097623
19.0894	1.098651
19.2775	1.099705
19.46559	1.100783
19.65369	1.101886
19.84178	1.103013
20.02987	1.104165
20.21797	1.105341
20.40606	1.106541
20.59416	1.107765
20.78225	1.109013
20.97035	1.110284
21.15844	1.111579
21.34653	1.112897

21.53463	1.114238
21.72272	1.115603
21.91082	1.11699
22.09891	1.1184
22.28701	1.119832
22.4751	1.121287
22.66319	1.122764
22.85129	1.124263
23.03938	1.125784
23.22748	1.127327
23.41557	1.128892
23.60367	1.130478
23.79176	1.132086
23.97986	1.133714
24.16795	1.135364
24.35604	1.137035
24.54414	1.138726
24.73223	1.140439
24.92033	1.142171
25.10842	1.143924
25.29652	1.145697
25.48461	1.14749
25.6727	1.149303
25.8608	1.151136
26.04889	1.152988
26.23699	1.15486
26.42508	1.156751
26.61318	1.158661
26.80127	1.16059
26.98936	1.162538
27.17746	1.164504
27.36555	1.166489
27.55365	1.168492
27.74174	1.170514
27.92984	1.172553
28.11793	1.17461
28.30603	1.176685
28.49412	1.178777
28.68221	1.180887
28.87031	1.183014
29.0584	1.185158
29.2465	1.187319
29.43459	1.189496
29.62269	1.191691

29.81078	1.193901
29.99887	1.196128
30.18697	1.19837
30.37506	1.200629
30.56316	1.202903
30.75125	1.205192
30.93935	1.207497
31.12744	1.209817
31.31553	1.212152
31.50363	1.214502
31.69172	1.216866
31.87982	1.219245
32.06791	1.221638
32.25601	1.224045
32.4441	1.226466
32.6322	1.228901
32.82029	1.231349
33.00838	1.23381
33.19648	1.236284
33.38457	1.238771
33.57267	1.241271
33.76076	1.243784
33.94886	1.246308
34.13695	1.248845
34.32504	1.251394
34.51314	1.253954
34.70123	1.256526
34.88933	1.259109
35.07742	1.261703
35.26552	1.264308
35.45361	1.266923
35.6417	1.269549
35.8298	1.272185
36.01789	1.274831
36.20599	1.277486
36.39408	1.280152
36.58218	1.282826
36.77027	1.285509
36.95837	1.288202
37.14646	1.290902
37.33455	1.293612
37.52265	1.296329
37.71074	1.299054
37.89884	1.301786

38.08693	1.304527
38.27503	1.307274
38.46312	1.310028
38.65121	1.312788
38.83931	1.315555
39.0274	1.318328

TABLE 2 Characteristics of Adjusted Variables After Propensity Matching.

	SIRI<11.24×10^9	SIRI>=11.24×10^9	p
N (sample size)	66	66	
gender (%)	13 (19.7)	17 (25.8)	0.533
Male			
Female	53 (80.3)	49 (74.2)	
Ethnicity (%)			0.327
White	21 (31.8)	29 (43.9)	
Black	2 (3.0)	1 (1.5)	
Other	43 (65.2)	36 (54.5)	
WBC,10^9g/l	14.2 (12.1, 16.2)	13.6 (11.7, 16.6)	0.955
Platelet, 10^9/L	190.0 (153.5, 233.0)	179.5 (137.5, 218.8)	0.386
Red blood cell, 10^9/L	3.9 (3.3, 4.4)	3.8 (3.3, 4.4)	0.740
Serum creatinine, mg/Dl	0.9 (0.8, 1.2)	0.8 (0.7, 1.1)	0.580
BUN, mg/dL	16.0 (12.0, 21.0)	17.0 (14.0, 23.0)	0.430
RDW, %	13.4 (12.9, 14.6)	13.5 (12.7, 14.6)	0.922
INR	1.2 (1.1, 1.3)	1.2 (1.1, 1.3)	0.991
PT, s	12.8 (11.6, 13.9)	12.8 (11.9, 13.9)	0.736
PTT, s	27.6 (25.4, 29.6)	27.3 (25.6, 29.1)	0.831
temperature, °C	37.2 (36.8, 37.6)	37.1 (36.9, 37.4)	0.610
RR, times/minute	19.0 (17.0, 21.0)	18.5 (17.3, 21.2)	0.566
SIRS	3.0 (2.0, 4.0)	3.0 (2.0, 3.0)	0.450
GCS	12.0 (7.2, 14.0)	13.0 (8.2, 14.0)	0.878
SOFA	5.0 (4.0, 8.0)	5.0 (3.0, 7.0)	0.437
APSIII	44.0(32.5,59.0)	44.0 (32.5, 52.0)	0.574
SAPSII	33.0(24.8, 38.8)	35.5 (26.2, 40.8)	0.425
